# Umbilical Cord Blood Testosterone and Childhood Internalizing and Externalizing Behavior: A Prospective Study

**DOI:** 10.1371/journal.pone.0059991

**Published:** 2013-04-01

**Authors:** Monique Robinson, Andrew J. O. Whitehouse, Peter Jacoby, Eugen Mattes, Michael G. Sawyer, Jeffrey A. Keelan, Martha Hickey

**Affiliations:** 1 Telethon Institute for Child Health Research, Centre for Child Health Research, The University of Western Australia, Perth, Australia; 2 School of Psychology, The University of Western Australia, Perth, Australia; 3 School of Paediatrics and Reproductive Health, University of Adelaide, Adelaide, South Australia; 4 Research and Evaluation Unit, Women’s and Children’s Network, Adelaide, South Australia; 5 School of Women’s and Infants’ Health, The University of Western Australia, Perth, Australia; 6 Department of Obstetrics and Gynaecology, University of Melbourne, Melbourne, Australia; CUNY, United States of America

## Abstract

Antenatal testosterone exposure influences fetal neurodevelopment and gender-role behavior in postnatal life and may contribute to differences in developmental psychopathology during childhood. We prospectively measured the associations between umbilical cord blood testosterone levels at birth and childhood behavioral development in both males and females from a large population based sample. The study comprised 430 females and 429 males from the Western Australian Pregnancy Cohort (Raine) Study where umbilical cord blood had been collected. Total testosterone concentrations were determined by mass spectrometry and bioavailable testosterone (BioT) levels were calculated. At two, five, eight and ten years of age, the participants completed the Child Behavior Checklist (CBCL). Linear regression models were used to analyse the relationship between BioT concentrations (in quartiles) and CBCL scores (total, internalizing, externalizing and selected syndrome). Boys had higher mean CBCL T-scores than girls across all ages of follow-up. There was no significant relationship between cord blood BioT quartiles and CBCL total, internalizing and externalizing T-scores at age two or five to ten combined. In the syndrome score analyses, higher BioT quartiles were associated with significantly lower scores for attention problems for boys at age five, eight and ten, and greater withdrawal symptoms in pre-school girls (age five). We did not identify a consistent relationship between antenatal testosterone exposure and total, internalizing or externalizing behavioral difficulties in childhood. Higher umbilical cord BioT levels were associated with lower scores for attention problems in boys up to 10 years and more withdrawn behavior in 5-year-old girls; however, these findings were not consistent across ages and require further investigation in a larger sample.

## Introduction

Both animal and human studies have reported that fetal androgen exposure during pregnancy can influence the postnatal development of sexually differentiated behaviors, with greater testosterone exposure thought to predispose to more masculine behaviors, such as aggression. In contrast, lower levels of intrauterine testosterone exposure are associated with more feminine behaviors, such as increased nurturing behavior [1], [2], [3]. However, there is relatively little evidence to inform the relationship between antenatal testosterone exposure and behavior in males and females from population based samples. There are clear differences in the nature of behavioral difficulties in childhood for boys and girls, with internalizing behaviors such as anxiety and withdrawal more common in girls, while externalizing behaviors such as aggression, impulsivity and conduct problems are more common in boys [4], [5]. The prenatal androgen environment may underlie these differences in behavior [6], but the evidence to support this remains limited.

The involvement of sex hormones in developmental psychopathology is supported by a number of small studies that have shown a greater exposure to testosterone *in utero* can lead to a shift in the behavior of females towards more male-typical behavior [7]. In a small cohort of 88 preschool girls, evidence of higher antenatal testosterone exposure measured by digit ratio was found to be associated with an increased risk for hyperactivity and poorer social functioning- behavioral problems usually more common in boys [8]. Another small study (n = 34) of females affected by congenital adrenal hyperplasia (CAH), an endocrine disorder characterised by the overproduction of fetal adrenal androgens, found increased expression of autistic traits (again, usually more common in boys than girls) compared with relatives unaffected by CAH [3].

Antenatal androgens may also influence behavior in childhood. Using digit ratio as a marker of antenatal androgen exposure, higher exposure was associated with increased externalising behavior in girls and boys at mid-childhood [6]. Antenatal testosterone exposure has also been associated with increased male-typical behavior in boys during middle childhood, although the effect was less pronounced than that observed in girls [9]. With regard to internalizing outcomes, exposure to higher levels of intrauterine testosterone was found to lead to increased fear reactivity, determined by the child’s reaction to an unfamiliar robotic toy, in infant boys but not in girls [10]. Higher salivary testosterone levels in male infants measured postnatally have also predicted increased negative affectivity, such as sadness, distress, and fear [11]. Conversely, exposure to lower levels of testosterone has been associated with an increase in emotional symptoms in boys in mid-childhood [8]. Findings are inconsistent, as some studies of internalizing disorders such as anxiety in males or females have failed to demonstrate an association with fetal androgen exposure [12].

The relationship between prenatal androgen exposure and childhood behavior has not previously been measured in large population based studies using biological measures of antenatal testosterone levels. The aim of this study was to use prospectively collected data from a non-selected pregnancy cohort in conjunction with umbilical cord blood testosterone measurements to measure the relationship between antenatal testosterone exposure and child behavior from age 2–10 years. Specifically, we hypothesised that females and males exposed to elevated testosterone levels *in utero* would have higher externalizing behavior scores (representing poorer behavior).

## Methods

### Participants

The Western Australian Pregnancy Cohort (Raine) Study commenced in May 1989 and recruited 2900 pregnancies at an average gestational age of 18 weeks through the state maternity hospital (King Edward Memorial Hospital; KEMH) until November 1991. The study was initially established to investigate the effects and safety of repeated ultrasound imaging in pregnancy. A full summary of the study and enrolment details has been published elsewhere [13]. Participants in the intensive ultrasound arm of the study provided blood samples and data regarding psychosocial and demographic characteristics at enrolment and again at 34 weeks’ gestation; neonates had umbilical cord blood sampled at delivery. Mothers and their children were followed up at age two, five, eight and ten years with a behavioral and physical health assessment. Informed written consent was obtained from the mother at enrolment and again at each follow-up. The protocols for the study were approved by the Human Research Ethics Committees at KEMH and/or Princess Margaret Hospital for Children in Perth, Western Australia.

### Attrition Bias

We were able to recruit 90% of eligible women approached to participate in this study and the initial cohort was representative of a tertiary hospital population [13]. Those who were socially disadvantaged were less likely to remain in the study in the early years; however, the sample was found to be comparable to the Western Australian population on various sociodemographic measures at later follow-ups [14]. At the 10-year follow-up, 2,047 study children and their families completed all or part of the follow-up (281 deferred participation, 162 were unable to be traced, 348 had withdrawn and 30 were deceased). This provided a response rate after 10 years of more than 70% of the original sample of 2,868 live born children.

### Child Behavior

The 118 item CBCL for Ages 4–18 (CBCL/4–18) was administered at the five, eight, and ten year follow-ups and completed by the primary caregiver (usually the mother) [15]. The 99-item Child Behavior Checklist for Ages 2–3 (CBCL/2–3), an empirically validated measure of child behavior by parent report, was used at the two-year follow-up [16]. The checklist includes a Total Behavior Problem Scale, which is comprised of all the behavioral items on the checklist, an Externalizing Scale which rates antisocial or under-controlled behavior, and an Internalizing Scale which rates inhibited or over-controlled behavior. In addition, there are eight subscales, which assess problems in more specific areas. These are labelled: withdrawn behavior, somatic complaints, anxiety/depression, delinquency, aggression, social, thought and attention problems [15]. On all scales, raw scores and T-scores (standardised by age and sex) are available. [15]. Higher scores indicate higher levels of emotional or behavioral problems. We compared the standardized T-scores with z-scores derived from the raw scores in our models, in case the T-scores were not a sensitive enough measure, but given there were no significant differences in our results we maintained the use of the T-scores in our analyses. Based on previous findings we selected four behavioral syndromes *a priori* as outcomes for additional analyses: aggression and attention problem raw scores (males) and withdrawn and social problem raw scores (females) [6], [8].

### Prenatal Androgen Exposure

Umbilical cord blood collected at birth was available from 861 randomly selected singleton deliveries. Cord blood samples consisted of mixed arterial–venous fetal blood. Lack of contamination was confirmed by Mendelian concordance analysis of DNA from matched maternal and cord blood in 10 randomly selected sample sets from the cohort using the Affymetrix genome-wide human single nucleotide polymorphisms array 6.0. Liquid chromatography–tandem mass spectrometry after solvent extraction was used to determine total testosterone concentrations, described elsewhere in detail by Keelan et al. [17]. Sex hormone binding globulin (SHBG) was measured by ELISA using a commercial kit (IBL International, Hamburg, Germany). Measurements of total testosterone, particularly during pregnancy when SHBG and albumin are increased, does not necessarily reflect biologically active testosterone levels [18]. Therefore, bioavailable testosterone (BioT), representing the fraction of total testosterone either free or bound to serum albumin, was calculated as previously described [17]. Albumin levels were adjusted to take into account the decrease in serum albumin concentrations with gestational age using published reference values [19]. BioT data were analysed in quartiles, with the lowest quartile designated as the reference category [20].

### Covariates

We adjusted for a range of antenatal factors that have previously been associated with later child behavioral outcomes [21]. These were alcohol consumption and cigarette smoking during pregnancy (measured at 34 weeks’ gestation), total family income, maternal age and maternal education, (measured at 18 weeks’ gestation). We also adjusted for type of labour onset (spontaneous or induced), parity, and gestational age at birth.

### Statistical Analyses

BioT and total testosterone levels were highly correlated (Spearman correlation coefficient 0.93 for girls and 0.93 for boys). We analysed mean T-scores separately for males and females at age two and five-ten years, adjusted for gestational age at delivery. Linear regression was used to investigate the relationship between BioT and total, internalizing and externalizing CBCL T-scores at ages two, five, eight and ten. CBCL scores at age 2 were analysed separately from the older age groups as the questionnaires differed slightly between the CBCL/2–3 and the CBCL/4–18. The combined CBCL outcomes at ages 5, 8 and 10 were analysed using longitudinal mixed regression models with a random intercept at the subject level to account for loss of independence due to repeated measures on the same individuals. In these longitudinal models, we checked that effects were consistent across ages by ensuring that BioT quartiles and age interaction effects were not statistically significant (p<0.05). To examine the relationship between BioT quartiles and specific syndromes, we used negative binomial regression (due to the skewed distribution of data) to analyse raw scores of four behavioral syndromes selected *a priori* at each follow-up. The syndrome scores included as outcomes in these models were: aggression and attention problems for males and social and withdrawn problems for females. Separate models were constructed for males and females and all models were adjusted for the covariates already noted: alcohol and smoking in pregnancy, parity, presence of labour, mother’s educational level, low family income, mother’s age (categorised as <20, 20–25, 25–30, 30–35 and >35) and gestational age at delivery.

## Results

Of the 861 available umbilical cord blood samples collected at birth, we had data for 430 females and 429 males. Median BioT concentrations were significantly lower in female children compared to males, *p*<0.001 (Table 1). There were no further significant differences observed between girls and boys for our control variables.

**Table 1:** Frequency characteristics of the sample predictor and control variables.

	Girls	Boys
	N = 430	N = 429
	n (%)	n (%)
**Alcohol in pregnancy**		
None	264 (61.4)	251 (58.5)
< = once a week	130 (30.2)	136 (31.7)
>once a week	21 (4.9)	25 (5.8)
missing	15 (3.5)	17 (4.0)
**Smoking in pregnancy**		
None	303 (70.5)	312 (72.7)
< = 10/day	65 (15.1)	52 (12.1)
>10/day	47 (10.9)	48 (11.2)
missing	15 (3.5)	17 (4.0)
**Low income (<24k/yr)**		
No	228 (53.0)	244 (56.9)
Yes	178 (41.4)	171 (39.9)
missing	24 (5.6)	14 (3.3)
**Mother’s education**		
> = yr12	147 (34.2)	155 (36.1)
<yr 12	266 (61.9)	263 (61.3)
missing	17 (4.0)	11 (2.6)
**Labour**		
No	47 (10.9)	56 (13.1)
Yes	366 (85.1)	362 (84.4)
missing	17 (4.0)	11 (2.6)
**Parity**		
1^st^ child	209 (48.6)	192 (44.8)
2^nd^ child	121 (28.1)	136 (31.7)
3^rd^+ child	99 (23.0)	101 (23.5)
missing	1 (0.2)	0
	**Mean (SD)**	**Mean (SD)**
**Maternal age** (years)	27.6 (5.9)	27.6 95.9)
**Gestational age at birth** (weeks)	273 (17)	274 (16)
	**Median (IQR)**	**Median (IQR)**
**Bioavailable T** (nmol/L)	0.07, (0.06)	0.12, (0.09)

**Figure 1:**
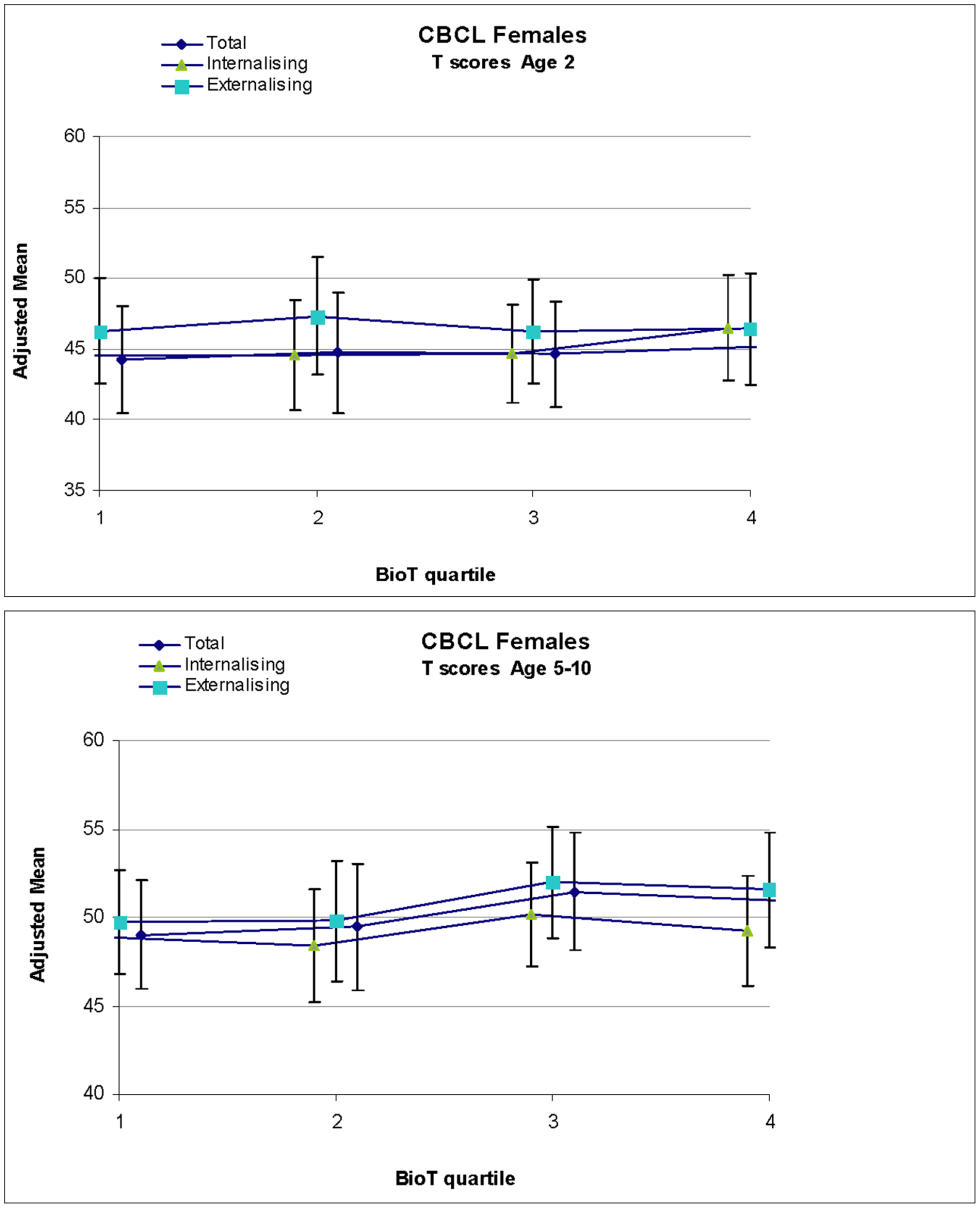
Mean CBCL total, internalizing and externalizing T-scores at age 2 and age 5–10 by BioT quartile for females.

**Figure 2:**
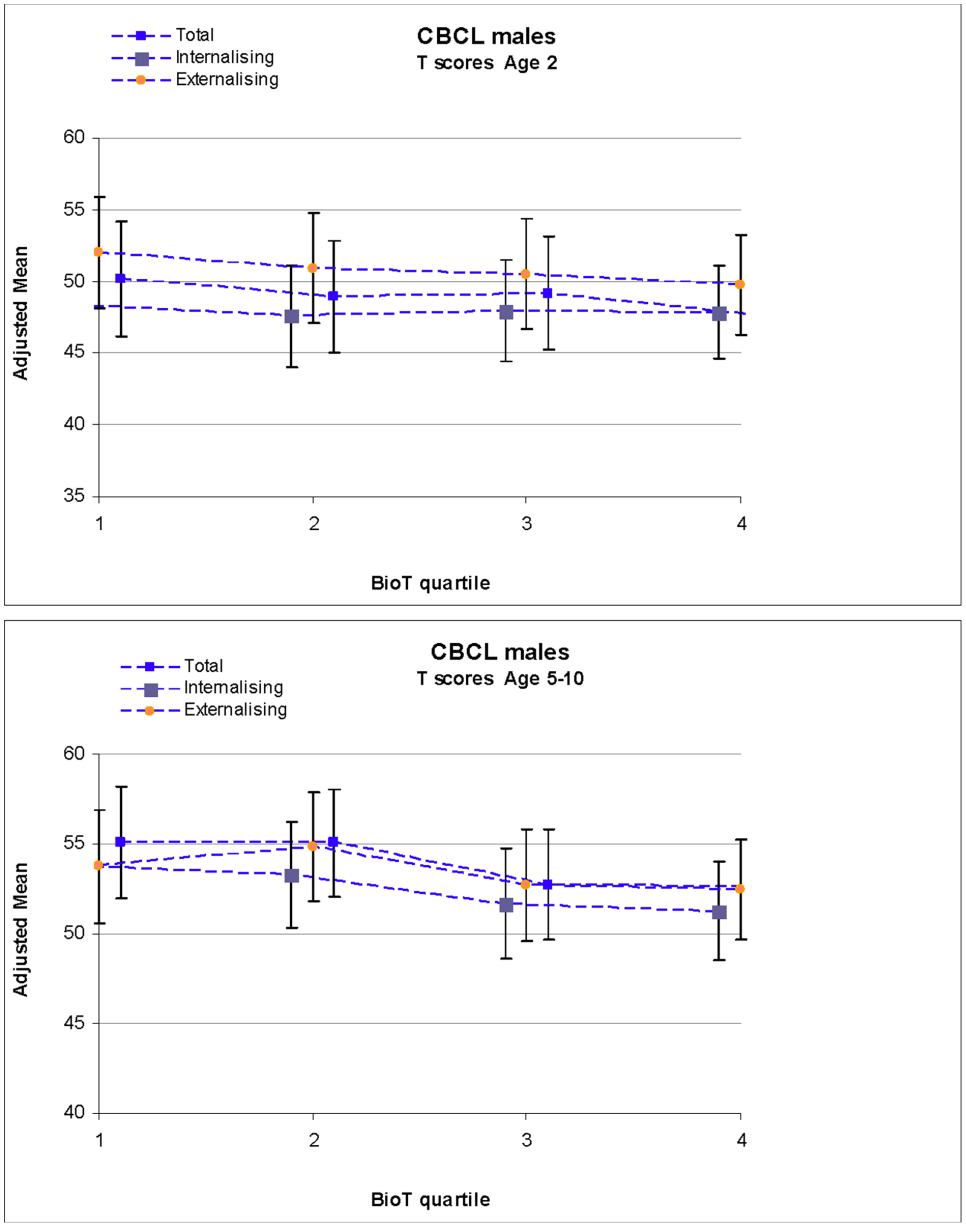
Mean CBCL total, internalizing and externalizing T-scores at age 2 and age 5–10 by BioT quartile for males.

Although the sex differences were non-significant, we observed generally higher mean T-scores (adjusted for gestational age only) in boys compared with girls in our cohort across total, internalizing and externalizing behavior at age two and five through ten years (Figure 1, Figure 2). In the fully adjusted linear regression model, higher BioT levels were not significantly associated with total CBCL T-scores for girls or boys at age two (Table 2). In comparison with the lowest quartile for BioT levels, the highest quartile of BioT level was associated with a non-significant increase in CBCL T-score of 0.97 in girls at age two, and a non-significant reduction in CBCL T-score of 2.58 in boys at age two. This pattern of non-significant sex differences in T-scores was similar for both internalizing and externalizing T-scores at age two, with boys with higher cord blood BioT levels showing lower internalizing (estimate of effects (EE) = −0.42, 95%CI = −3.91, 3.07) and externalizing scores (EE = −2.24, 95%CI = −6.03, 1.54), while girls with the highest BioT levels had higher internalizing (EE = 1.90, 95%CI = −1.46, 5.26) and externalizing (EE = 0.14, 95%CI = −3.41, 3.68) scores. In the random effects model presenting the CBCL outcomes from age five to ten years combined (Table 3), we did not find a significant association between BioT quartile and CBCL T-scores.

**Table 2:** Linear regression model of prenatal bioavailable testosterone (BioT) levels on CBCL T-scores at age 2 years.

	Total			Internalizing			Externalizing		
BioT quadrant comparison	Effect Size^a^	95%CI	p-value	Effect Size^a^	95%CI	p-value	Effect Size^a^	95%CI	p-value
**Girls Q2 v Q1**	0.49	−3.19, 4.18	0.37	−0.02	−3.42, 3.38	0.66	1.06	−2.53, 4.65	0.33
** Q3 v Q1**	0.41	−3.22, 4.05		0.08	−3.27, 3.44		−0.06	−3.60, 3.48	
** Q4 v Q1**	0.97	−2.66, 4.61		1.90	−1.46, 5.26		0.14	−3.41, 3.68	
**Boys Q2 v Q1**	−1.23	−4.74, 2.28	0.14	−0.69	−3.85, 2.47	0.23	−1.06	−4.48, 2.36	0.33
** Q3 v Q1**	−0.97	−4.52, 2.58		−0.33	−3.52, 2.87		−1.47	−4.94, 1.99	
** Q4 v Q1**	−2.58	−5.50, 2.28		−0.42	−3.91, 3.07		−2.24	−6.03, 1.54	

Longitudinal mixed model adjusted for gestational age, alcohol use and smoking during pregnancy, mother’s education, low family income, parity, mother’s age and presence of labour.

^a^Effect size refers to the difference between the adjusted mean T-score for each quartile and the reference quartile.

**Table 3:** Random effects model effect of prenatal bioavailable testosterone (BioT) levels on CBCL T-scores at age 5–10 years.

	Total			Internalizing			Externalizing		
BioT quadrant comparison	Effect Size^a^	95%CI	p-value	Effect Size^a^	95%CI	p-value	Effect Size^a^	95%CI	p-value
**Girls Q2 v Q1**	0.45	−2.66, 3.56	0.96	−0.52	−3.32, 2.28	0.61	0.04	−2.95, 3.02	0.92
** Q3 v Q1**	2.43	−0.66, 5.52		1.27	−1.51, 4.05		2.25	−0.72, 5.21	
** Q4 v Q1**	1.93	−1.24, 5.11		0.33	−2.53, 3.19		1.79	−1.25, 4.84	
**Boys Q2 v Q1**	−0.03	−2.67, 2.62	0.63	−0.54	−3.18, 2.08	0.98	1.09	−1.57, 3.75	0.69
** Q3 v Q1**	−2.36	−5.12, 0.41		−2.13	−4.86, 0.61		−1.02	−3.80, 1.76	
** Q4 v Q1**	−2.44	−5.33, 0.45		−2.53	−5.39, 0.33		−1.31	−4.21, 1.60	

Longitudinal mixed model adjusted for gestational age, alcohol use and smoking during pregnancy, mother’s education, low family income, parity, mother’s age and presence of labour.

^a^Effect size refers to the difference between the adjusted mean T-score for each quartile and the reference quartile.

We also did not find any significant relationship between cord BioT levels and aggression problems in boys (Table 4) or social problems in girls (Table 5). We did observe significant rate ratios linking higher BioT levels with higher withdrawn behavior raw scores for girls at age five, but this relationship lost significance at age eight and ten years. At age five, eight and ten years, boys in the highest BioT quartiles (Q3 and/or Q4 v Q1) were observed to have significantly lower attention problem raw scores following adjustment for all covariates.

**Table 4:** Modeled effect of prenatal bioavailable testosterone (BioT) levels on raw syndrome scores- males.

	Aggression problems			Attention problems*		
BioT quadrant comparison	Effect size^a^	95%CI	p-value	Effect size^a^	95%CI	p-value
Age 2 Q2 v Q1	0.92	0.74, 1.13	0.73			
Q3 v Q1	0.91	0.74, 1.14				
Q4 v Q1	0.88	0.70, 1.12				
Age 5 Q2 v Q1	1.04	0.83, 1.29	0.21	1.02	0.79, 1.33	0.053
Q3 v Q1	0.82	0.65, 1.03		0.73	0.55, 0.97	
Q4 v Q1	0.91	0.72, 1.16		0.82	0.61, 1.10	
Age 8 Q2 v Q1	1.00	0.78, 1.28	0.61	1.04	0.78, 1.40	0.048
Q3 v Q1	1.05	0.81, 1.35		0.69	0.51, 0.95	
Q4 v Q1	0.87	0.67, 1.14		0.81	0.59, 1.11	
Age 10 Q2 v Q1	0.99	0.74, 1.32	0.28	0.83	0.59, 1.17	0.008
Q3 v Q1	0.92	0.68, 1.24		0.58	0.40, 0.83	
Q4 v Q1	0.75	0.55, 1.03		0.61	0.42, 0.88	

Cross-sectional negative binomial models adjusted for gestational age, alcohol use and smoking during pregnancy, mother’s education, low family income, parity, mother’s age and presence of labour.

* Attention problems not measured at age 2.

^a^Effect size refers to the ratio of adjusted raw scores between each quartile and the reference quartile.

**Table 5:** Modeled effect of prenatal bioavailable testosterone (BioT) levels on raw syndrome scores- females.

	Social problems*			Withdrawn behavior		
BioT quadrant comparison	Effect size^a^	95%CI	p-value	Effect size^a^	95%CI	p-value
Age 2 Q2 v Q1				1.05	0.79, 1.41	0.49
Q3 v Q1				0.95	0.71, 1.27	
Q4 v Q1				1.18	0.89, 1.57	
Age 5 Q2 v Q1	1.14	0.83, 1.56	0.52	1.63	1.18, 2.26	0.008
Q3 v Q1	1.26	0.93, 1.70		1.59	1.15, 2.19	
Q4 v Q1	1.13	0.81, 1.56		1.58	1.13, 2.21	
Age 8 Q2 v Q1	1.18	0.82, 1.69	0.57	1.14	0.80, 1.62	0.22
Q3 v Q1	1.28	0.90, 1.82		1.24	0.88, 1.75	
Q4 v Q1	1.10	0.76, 1.61		1.11	0.77, 1.60	
Age 10 Q2 v Q1	0.90	0.58, 1.39	0.96	1.11	0.76, 1.61	0.88
Q3 v Q1	0.93	0.61, 1.42		0.95	0.66, 1.38	
Q4 v Q1	0.97	0.63, 1.51		1.07	0.73, 1.56	

Cross-sectional negative binomial models adjusted for gestational age, alcohol use and smoking during pregnancy, mother’s education, low family income, parity, mother’s age and presence of labour.

* Social problems not measured at age 2.

^a^Effect size refers to the ratio of adjusted raw scores between each quartile and the reference quartile.

## Discussion

This is the first published study to report the relationship between cord blood BioT concentrations and male and female childhood behavior in a large population-based sample. Contrary to previous smaller studies suggesting that greater exposure to testosterone *in utero* significantly impacts upon child internalizing and externalizing behavior [3], [6], [8], [10], we found no significant associations between BioT concentrations and changes in behavior as measured by total, internalizing and externalizing T-scores from age two through to middle childhood. However, when we assessed sub-scale specific syndromes, we observed that boys in the higher BioT quartiles had significantly better attention behavior scores from ages five through ten years, while girls had significantly higher withdrawn behavior scores, but only at age five. While causation cannot be determined from these results alone, they do represent an important area of investigation for future research.

Overall, our findings indicate that fetal testosterone concentrations in late gestation do not predict an increased risk for behaviour problems in childhood in boys or girls. Previous studies have reported significantly higher rates of childhood problem externalizing and internalizing behaviors associated with prenatal testosterone exposure. However, these studies have been limited by population selection [3], small sample sizes [6], [8] or single point outcome measures [10], [11] [8]. In contrast, our study uses data from four time points from age two to ten years from a relatively large population-based cohort.

Our finding regarding improved attention with higher fetal testosterone levels at birth may contribute to the understanding of the mechanisms responsible for the gender differences in attention problems, where boys are less likely than girls to have the inattentive subtype of ADHD [22]. A study of newborn male chicks found that testosterone administration decreased distractibility compared with controls [23], and research on older adults found that as testosterone declined, performance on memory and speed cognitive tests improved [24]. However, this contrasts with a previous study of older male adults that found that lower levels of testosterone were associated with slower processing speed and poorer executive function, and another study of males and females aged 18–77 that found higher androgen levels led to increased attention [25], [26]. Our finding that girls with higher cord BioT levels, presumably reflecting greater testosterone exposure *in utero*, had higher withdrawal behavior problem scores at age five may reflect a similar phenomenon underpinning the difficulties in communication and social behavior for girls with CAH that have been described in previous work [3].

The strength of this study is the size of the cohort and the prospective ten year longitudinal follow-up of behavioral development using a valid and detailed assessment instrument [27]. We had high rates of retention with over 70% of our original birth cohort still participating at ten years. We used umbilical cord blood as an indirect biological measure of fetal testosterone exposure, allowing us to assess differences at a general population level as opposed to being limited to groups of individuals with clinical endocrine disorders [3], [28]. Testosterone was measured using a highly sensitive, selective and accurate assay to avoid interference by the numerous other steroid hormones known to be present in cord blood. While all surrogate measures are vulnerable to a lack of concordance between the measured endpoint and the assumed biological correlate [29], our findings using umbilical cord blood are similar to those observed using amniotic fluid in the second trimester to measure prenatal testosterone exposure [30]. In this study our aim was to examine behavioral outcomes in childhood and we deliberately focused on pre-pubertal development, prior to significant changes in endogenous sex steroid concentrations.

A potential limitation of our study is the use of testosterone measures obtained during late gestation. The sensitive period(s) when testosterone may affect fetal brain development are not known, but sex specific changes have been documented from early gestation. Measurements during late gestation may not necessarily reflect earlier exposure. Further, testosterone effects on the brain may not relate solely to circulating levels, but may also reflect receptor sensitivity which cannot be easily measured in human studies. We acknowledge that although we did include a range of maternal sociodemographic and lifestyle factors from pregnancy in our models as potential confounders, there are potentially other factors later in childhood that remain unmeasured and unaccounted for in this study. However, despite these limitations and our inability to determine causation from this study alone, our study of the relationship between late gestation umbilical cord testosterone levels and childhood behavior adds significantly to the existing literature in this area.

In summary, our findings did not confirm the hypothesis that antenatal testosterone exposure is a significant determinant of later internalizing or externalizing child behavioral problem scores. However, we did observe significantly lower attention problem scores from age five to ten in boys with higher cord blood BioT levels and higher withdrawn problem scores at age five in girls. Further research is warranted to more thoroughly understand this relationship, but these findings suggest that the links between intrauterine testosterone exposure and behavioral development may be restricted to particular behaviors.
